# Design, optimization, and ADMET evaluation of S11a-0000168202: A promising LIMK1 inhibitor for gastric cancer treatment

**DOI:** 10.1371/journal.pone.0323699

**Published:** 2025-05-14

**Authors:** Guojun Li, Jionghuang Chen, Rui Chen, Weihua Yu

**Affiliations:** 1 Department of General Surgery, Shangyu People’s Hospital of Shaoxing, Shaoxing University, Shaoxing, China; 2 Department of General Surgery, Sir Run Run Shaw Hospital, School of Medicine, Zhejiang University, Hangzhou, China; 3 College of Life Sciences and Health Engineering, Jiangnan University, Wuxi, China; Kwara State University, NIGERIA

## Abstract

This study focuses on the development and optimization of S11a-0000168202, a novel LIMK1 inhibitor with potential therapeutic applications in gastric cancer. Through scaffold hopping and structural modification of HIT100844099, S11a-0000168202 demonstrated enhanced binding stability and stronger interactions with key LIMK1 residues, including GLU-414, ILE-416, and HIS-464. Molecular dynamics simulations and MMGBSA analyses confirmed the compound’s stability, while ADMET evaluation revealed favorable properties such as moderate lipophilicity, good human intestinal absorption, and low P-glycoprotein inhibition. Despite the promising computational results, the lack of experimental validation remains a limitation. Future studies should focus on in vitro and in vivo testing to confirm S11a-0000168202’s efficacy, pharmacokinetics, and safety. This compound holds significant potential as a therapeutic agent for LIMK1-targeted gastric cancer treatment.

## 1. Introduction

Gastric cancer is a major malignancy with high global incidence and mortality rates. According to the latest World Health OrganizaAtion (WHO) statistics for 2024, there were approximately 968,400 new cases of gastric cancer worldwide in 2022, accounting for 4.9% of all cancer cases, ranking fifth among all cancers in incidence. Additionally, gastric cancer caused an estimated 659,900 deaths in 2022, making it the fourth leading cause of cancer-related deaths globally [1]. This high prevalence is attributed to factors such as genetic susceptibility, Helicobacter pylori infection, dietary habits (e.g., high salt intake and consumption of pickled foods), smoking, and environmental exposures [2]. Although recent advancements in gastric cancer treatment, such as surgery, radiotherapy, chemotherapy, and targeted therapy, have improved outcomes, most patients are diagnosed at an advanced stage, leading to poor overall survival rates [3]. Therefore, there is an urgent need to develop more effective treatments to improve patient prognosis and quality of life.

Targeted therapies have transformed the treatment landscape for gastric cancer, with HER2 inhibitors (e.g., trastuzumab) [4] and immune checkpoint inhibitors (e.g., pembrolizumab) representing major advancements [5]. HER2 inhibitors specifically target tumors with HER2 overexpression, which is present in approximately 15–20% of gastric cancer patients [6]. These therapies have shown improved outcomes in this subset but are ineffective in HER2-negative patients. Immune checkpoint inhibitors, such as PD-1/PD-L1 inhibitors, enhance antitumor immunity and have demonstrated efficacy in advanced or metastatic gastric cancer, particularly in patients with high microsatellite instability (MSI-H) or high tumor mutational burden (TMB)[7]. However, these therapies are limited by patient selection, the potential for immune-related adverse events, and acquired resistance.

LIM kinase 1 [8] is a serine/threonine kinase that regulates cytoskeletal dynamics and plays a crucial role in cell migration, invasion, and tumor progression [9]. In recent years, abnormal expression and activation of LIMK1 have been widely reported in various cancers, particularly in gastric cancer [10], where high LIMK1 expression is closely associated with tumor aggressiveness and poor prognosis [11]. Thus, LIMK1 has emerged as a promising molecular target for gastric cancer treatment. Several LIMK1 inhibitors, such as LIMKi3, have been reported, showing good anti-tumor activity in vitro; however, their clinical applications face challenges such as selectivity and pharmacokinetic optimization [12]. Therefore, further exploration and optimization of LIMK1 inhibitors are crucial for the treatment of gastric cancer.

Studies have shown that LIMK1 is frequently upregulated in various cancers, including gastric cancer, and its overexpression is closely associated with enhanced tumor aggressiveness, lower survival rates, and poor prognosis. In contrast, the expression level of LIMK2 in gastric cancer is relatively lower, and its specific role has not been fully elucidated. The distinct overexpression of LIMK1 in gastric cancer makes it a more attractive therapeutic target compared to LIMK2. They phosphorylate and inactivate cofilin, leading to increased actin polymerization [13]. LIMK1 not only plays a critical role in cytoskeletal regulation but also contributes to tumor progression by affecting cell migration, proliferation, and invasion [10]. Specifically, LIMK1 facilitates the migration and invasion of tumor cells by remodeling the cytoskeleton, a process that is essential for tumor metastasis [14]. Furthermore, LIMK1 may promote angiogenesis, thereby supporting tumor growth and metastasis by enhancing blood supply [15]. Additionally, LIMK1 may influence immune cells within the tumor microenvironment, contributing to immune evasion [16]. Studies suggest that LIMK1 regulates immune cell recruitment and function, potentially suppressing the host’s immune response and promoting immune escape, thus facilitating tumor progression [17]. Consequently, LIMK inhibitors have emerged as a promising treatment strategy for certain cancers and neurological disorders. To better understand the role of these kinases in health and disease, high-quality chemical probes are required. Previous studies have conducted comparative evaluations of 17 LIMK1/2 inhibitors in various in vitro enzymatic and cellular assays, identifying three compounds (TH-257, LIJTF500025, and LIMKi3) as potent and selective inhibitors suitable for in vitro and in vivo studies of LIMK function [18]. Additionally, LIMK inhibitors are considered potential therapeutic agents for various indications, including elevated intraocular pressure (IOP), cancer, and HIV-1 infection, with LX-7101 advancing to Phase I clinical trials for glaucoma treatment [19]. Studies based on the design, synthesis, and evaluation of LIMK inhibitors using a pyrrolopyrimidine scaffold have revealed effective inhibition of LIMK1 and LIMK2, as well as inhibition of ROCK2 and PKA, by LX-7101 and its analogs.

Computer-Aided Drug Design (CADD) is a strategy that combines computational simulations with experimental validation and has been widely applied in drug discovery, particularly in the identification and optimization of targeted small molecule inhibitors [20]. In gastric cancer treatment, traditional chemotherapy regimens have significant limitations, including high toxicity and poor selectivity. CADD can accelerate the discovery of efficient and low-toxicity targeted inhibitors through virtual screening, molecular docking, and molecular dynamics simulation, thereby providing a new approach for personalized treatment of gastric cancer [21].

This study aims to utilize CADD to identify and optimize potential targeted inhibitors against LIMK1 for the treatment of gastric cancer. First, we will conduct virtual screening to identify candidate compounds that can bind to LIMK1. Then, molecular dynamics simulations will be used to analyze the stability and interaction modes of these candidate compounds with LIMK1 to ensure effective inhibition of the target. Additionally, scaffold hopping will be applied to modify the structure of the candidate compounds to optimize their efficacy and pharmacokinetic properties. Through these approaches, we hope to gain deeper insight into the molecular mechanisms of LIMK1 inhibition, providing theoretical support and candidate compounds for further drug development. This research aims to provide a scientific basis for LIMK1-targeted gastric cancer therapy, potentially improving treatment outcomes and patient quality of life.

## 2. Method

### 2.1 Preparation of LIMK1 structure and redocking of LIMKi3

Obtained from the Protein Data Bank, the crystal structure of LIMK1 with the reference inhibitor LIMKi3 (PDB ID 8AAU) [22] displays a resolution of 1.74 Å. Through the Schrodinger suite’s protein preparation wizard [23] and prime module, hydrogen atoms are added, missing loops filled, termini capped, charge states adjusted, and inappropriate H-bond orders fixed. Various steric strains and heavy atoms up to 0.3 Å were eliminated using the restrained energy minimization OPLS4 force field. LIMKi1, sourced from PubChem, underwent preparation with the ligprep module in Schrodinger Maestro for docking validation. Docking of the prepared ligand into the active binding sites was performed using the Glide protocol in the Schrödinger suite. The docking process considered key residues within the binding pocket, including GLY-480, ASP-460, VAL-476, TYR-415, GLY-348, GLY-419, LEU-467, LEU-382, PHE-350, GLY-418, THR-380, ALA-477, VAL-366, LYS-355, ILE-354, GLU-414, ILE-416, ARG-469, GLU-369, ILE-412, PHE-381, PHE-479, SER-463, GLU-343, ASN-512, GLY-351, LEU-345, CYS-349, GLU-470, GLU-376, LYS-347, MET-367, PHE-411, VAL-468, THR-413, THR-420, ASN-465, GLU-384, HIS-464, ALA-353, LEU-370, LYS-368, CYS-466, ASP-478, MET-388, LEU-397, VAL-475, ASN-462, GLN-352, VAL-344, LYS-417, TRP-515, LEU-481, THR-377, and GLY-346.

### 2.2 Compound Library Preparation

Accessed from the freely available TargetMol database (https://www.targetmol.cn/), a library containing 1.64 million compounds was acquired. Using the LigPrep tool [24], the ligand molecules were prepared, followed by geometric minimization employing the OPLS4 force field [25], ensuring specified chirality is retained at a pH range of 7.0 ± 2.0. The filtering criteria applied during compound library preparation, including compliance with Lipinski’s Rule of Five and additional parameters such as the number of rotatable bonds.

### 2.3 Structure-based Virtual Screening

To specify the docking site, the receptor grid generation panel was used to generate a grid around the co-crystallised ligand within the protein, with a grid size of 20 Å. The Glide ligand docking panel of Schrödinger Suite 2023 was used to create and manage docking jobs. In virtual screening workflow pre-screened ligands from the phase screening were subjected to standard precision flexible ligand docking and top 10% scored molecules were further subjected to extra precision (XP) flexible ligand docking. During XP docking Prime/MM-GBSA free energy [26] was calculated for top 10% molecules using the default configuration of the Prime module by Schrödinger using the protein-ligand complexes.

### 2.4 Structural Similarity Calculations between Top 10% XP Compounds and LIMKi3

LIMKi3 was used as a reference molecule, and Schrodinger’s Shape Screening tool [27] was employed for screening. This involved two main types of similarity calculations: shape similarity and chemical feature similarity. Shape similarity was assessed using the ROCS algorithm [28], which quantified shape matches based on the overlay of molecular volumes; chemical feature similarity considered functional groups such as hydrogen bond acceptors, donors, and hydrophobic regions, using chemical feature mapping techniques.

### 2.5. ADME and toxicity properties of selective compounds

Following that, we used the ADMETlab 2.0 web tool to assess the properties of the studied compounds for ADME and toxicity (https://admetmesh.scbdd.com/). In terms of absorption and distribution, the blood–brain barrier (BBB), human intestinal absorption (HIA), Caco-2 permeability, P-glycoprotein substrate, and subcellular localization were assessed. For metabolism, various common CPYP450 isoforms related to xenobiotic metabolism (CYP1A1, CYP1A2, CYP2C9, CYP2D6, CYP2C19, and CYP3A4) were examined. Probability proportions (%) were used to represent all predictions. For toxicity, AMES toxicity, carcinogens, acute oral toxicity, fish toxicity (median lethal concentration (pLC) 50, mg/L), and rat acute toxicity (lethal dose (LD) 50, mol/kg) were considered.

### 2.6. Molecular dynamics simulations

In the initial phase, all-atom MD simulations were conducted using the Desmond module of the Schrödinger suite within Maestro. Starting with docked complexes in a 10 Å buffered cubic water box, simulations incorporated the SPC water model [29] and 0.15 M NaCl for physiological accuracy. Electrostatic and van der Waals interactions were managed using the particle-mesh Ewald method and a 9.0 Å cutoff, respectively [30]. After solvation, minimization and equilibration followed using Desmond’s default protocol in both NVT and NPT ensembles. A 100 ns MD simulation in the NPT ensemble was completed with periodic boundary conditions using the OPLS4 force field. Temperature and pressure were controlled at 300 K and 1 atm using the Nosè-Hoover chain thermostat and the Martyna-Tobias-Klein barostat, respectively. The initial phase led to a multi-step protocol, starting with Brownian dynamics at 10 K and progressing through various restrained and unrestrained NVT and NPT simulations, culminating in a 500 ns NPT simulation maintaining the same temperature and pressure conditions.

## 3. Results

### 3.1 Validation of LIMKi3 Re-Docking to LIMK1 evaluating conformational consistency across different docking accuracy levels

**Fig 1:**
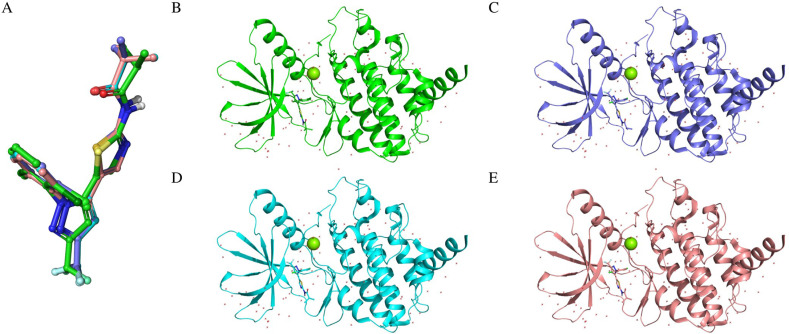
Evaluation of re-docking accuracy across different docking precision levels. **(A)** Superimposition of the LIMKi3 binding conformations obtained from three different accuracy levels with the co-crystal structure. **(B)** Binding mode of LIMKi3 in the co-crystal state with LIMK1. **(C)** Binding mode of LIMKi3 with LIMK1 obtained from HTVS accuracy calculation. **(D)** Binding mode of LIMKi3 with LIMK1 obtained from SP accuracy calculation. **(E)** Binding mode of LIMKi3 with LIMK1 obtained from XP accuracy calculation.

To establish LIM Domain Kinase 1 (LIMK1) bound to LIMKi3 (PDB ID 8AA as the reference structure, a docking box was constructed around LIMKi3. LIMKi3 was then dissociated from LIMK1 and re-docked using three different accuracy levels: Glide HTVS(High-Throughput Virtual Screening), Glide SP(Standard Precision), and Glide XP(Extra Precision). The docking results were evaluated for conformational consistency with the crystal structure by calculating the RMSD. Alignments of the original LIMKi3 conformation with the docked conformations from each accuracy level revealed near-perfect overlap in the core region, as shown in Fig 1A, with slight deviations only in the terminal groups. The RMSDs for HTVS, SP, and XP were 0.544 Å, 0.536 Å, and 0.553 Å, respectively. These values are well below the generally accepted threshold of 2 Å for successful redocking, indicating that the docking process reliably reproduced the original binding pose of LIMKi3. This high accuracy in redocking provides confidence in the validity of the docking methodology employed in this study.

Further analysis incorporating the LIMK1 structure, as depicted in Figs 1B–E, demonstrated that the conformations obtained through the three docking methods were nearly identical to the crystal structure. These results confirm the reliability of the virtual screening process using HTVS, SP, and XP accuracies, supported by the consistency of the re-docking outcomes.

### 3.2. Virtual Screening and Filtering of LIMK1 inhibitors identified from 1.64 million compounds

After validating the re-docking process, virtual screening was performed on 1.64 million compounds from TargetMol using the three validated accuracy methods. The compounds were evaluated based on State Penalty and MMGBSA, with their similarity to LIMKi3 also assessed. Due to the large number of compounds and limited computational resources, filtering thresholds were applied: State Penalty set to 0, docking score below -10, and MMGBSA less than -50 kcal/mol. As summarized in Table 1, 11 compounds met these criteria. All had a State Penalty of 0, docking scores ranging from -10.006 to -10.985, and MMGBSA values below -50 kcal/mol.

**Table 1:** Evaluation results of screened compounds.

hit_id	State Penalty	docking score	MMGBSA dG Bind	SMILES
HIT102568167	0	‒10.106	–57.92	CCOc1ccc(-c2n[nH]c3c2[C@@H](c2ccccc2F)C(C#N)=C(N)O3)cc1OC
HIT103799421	0	–10.172	–57.22	COc1ccc(-c2n[nH]c3c2[C@@H](c2ccccc2F)C(C#N)=C(N)O3)cc1OC
HIT103203505	0	–10.02	–55.44	COc1cccc([C@H]2C(C#N)=C(N)Oc3[nH]nc(-c4cccc([N+](=O)[O-])c4)c32)c1
HIT101601533	0	–10.056	–55.14	CC(C)Cc1ccc(-c2n[nH]c3c2[C@@H](c2cccs2)C(C#N)=C(N)O3)cc1
HIT100835212	0	–10.135	–54.69	COc1cccc([C@H]2C(C#N)=C(N)Oc3[nH]nc(-c4ccc(CC(C)C)cc4)c32)c1
HIT105357965	0	–10.106	–53.71	COc1cc(-c2n[nH]c3c2[C@@H](c2c(F)cccc2Cl)C(C#N)=C(N)O3)cc(OC)c1OC
HIT102689075	0	–10.006	–53.58	COc1ccc(OC)c([C@H]2C(C#N)=C(N)Oc3[nH]nc(-c4ccc(Cl)c(Cl)c4)c32)c1
HIT107134831	0	–10.046	–53.09	Cc1ccccc1Nc1nccc([C@H]2CCCN(C(=O)CC3CCCCC3)C2)n1
HIT102031352	0	–10.04	–52.9	COc1ccccc1[C@H]1C(C#N)=C(N)Oc2[nH]nc(-c3ccc(Cl)c(Cl)c3)c21
HIT101337631	0	–10.044	–52.25	COc1cccc([C@H]2C(C#N)=C(N)Oc3[nH]nc(-c4ccc(Cl)c(Cl)c4)c32)c1OC
HIT100844099	0	–10.985	–50.1	COc1cc([C@H]2C(C#N)=C(N)Oc3[nH]nc(-c4ccncc4)c32)ccc1O

**Fig 2:**
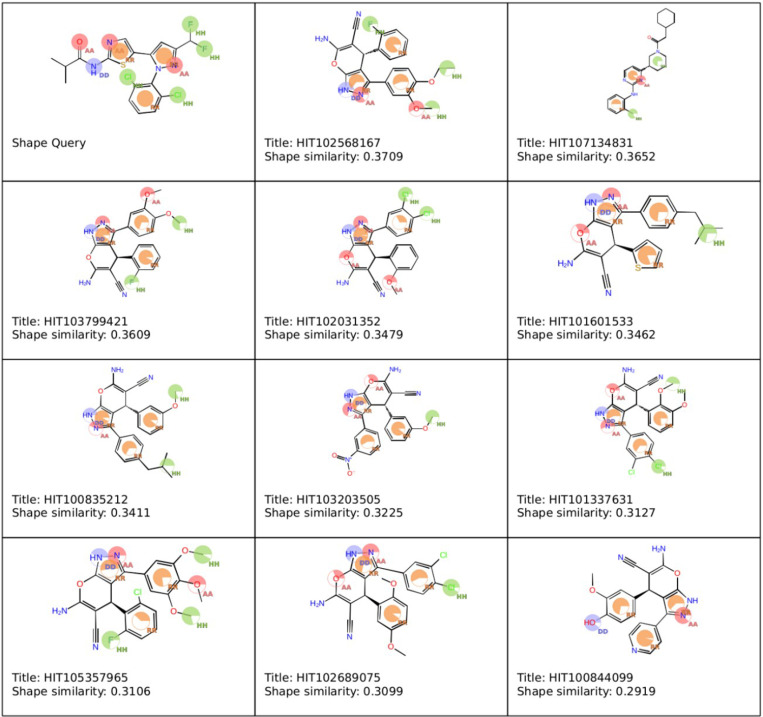
Pharmacophore features and similarity scores of different compounds. Base feature spheres for hydrogen bond acceptors (pink), donors (blue), hydrophobes [31], and aromatic rings (orange).

In the subsequent similarity analysis (Fig 2), the similarity between the identified compounds and LIMKi3 was generally low, ranging from 0.2919 to 0.3709. This suggests that, while these compounds may interact well with the binding site, their structural divergence from LIMKi3 could introduce novel binding modes or mechanisms of inhibition. As a result, these compounds demonstrate promising potential as LIMK1 inhibitors. However, their unique structural features warrant further investigation, particularly through experimental validation, to confirm their efficacy and explore their binding mechanisms in greater detail.

### 3.3. ADMET Evaluation of HIT100844099 and HIT103203505 Compared to LIMKi3

**Fig 3:**
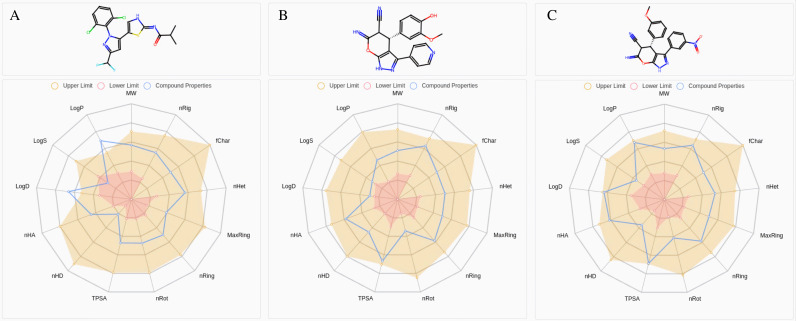
Medicinal Chemistry Radar Chart. **(A)** LIMKi3 **(B)** HIT100844099 **(C)** HIT103203505.

In preparation for interaction analysis, ADMET assessments were conducted comparing the physicochemical properties of HIT100844099 and HIT103203505 with LIMKi3, as illustrated in Fig 3 and S1 Fig. Both compounds outperformed LIMKi3 in key ADMET parameters, suggesting superior biopharmaceutical potential. For instance, their LogS values of ‒3.540 and ‒3.124 (compared to ‒5.986 for LIMKi3) indicate better solubility. Their lower LogD and LogP values—1.195, 2.908 and 1.320, 2.826 respectively, compared to 4.468 and 4.724—suggest more balanced distribution and reduced hydrophobicity. Additionally, they possess more protonatable acceptor and donor atoms, facilitating biomolecular interactions. Surface polar areas of 127.9 and 137.92 (vs. 63.04 for LIMKi3) further improve solubility and cellular permeability. Both compounds also show greater molecular complexity due to more ring structures and rigid bonds.

Medicinal chemistry evaluations (S1 Table) indicate higher human intestinal absorption (HIA) rates for these compounds—0.02 and 0.037 compared to 0.003 for LIMKi3. HIT103203505 shows particular promise in bioavailability (F(20%) and F(30%)), suggesting enhanced outcomes in vivo. Notably, HIT103203505 inhibits CYP2D6 significantly (0.674), a crucial enzyme in drug metabolism, while HIT100844099 exhibits lower inhibitory activity against CYP1A2 and CYP2C19, minimizing drug interaction risks.

Both compounds also excel in Caco-2 permeability, blood-brain barrier penetration, plasma protein binding (PPB), and unbound drug fraction (Fu). HIT100844099 shows a higher Fu (22.09% vs. 1.76% for LIMKi3), implying better physiological activity. Although both compounds exhibit slightly elevated hERG channel inhibitory activity, it remains within safe limits. Their compliance with Pfizer, GSK standards, and the Rule of Three underscores their broader drug development potential.

In summary, HIT100844099 and HIT103203505 demonstrate clear advantages in absorption, metabolism, and drug design flexibility, making them strong candidates for preclinical evaluation, balancing efficacy and safety.

### 3.4. Interaction Analysis of LIMKi3 HIT100844099 and HIT103203505 with LIMK1 Key Residues

**Fig 4:**
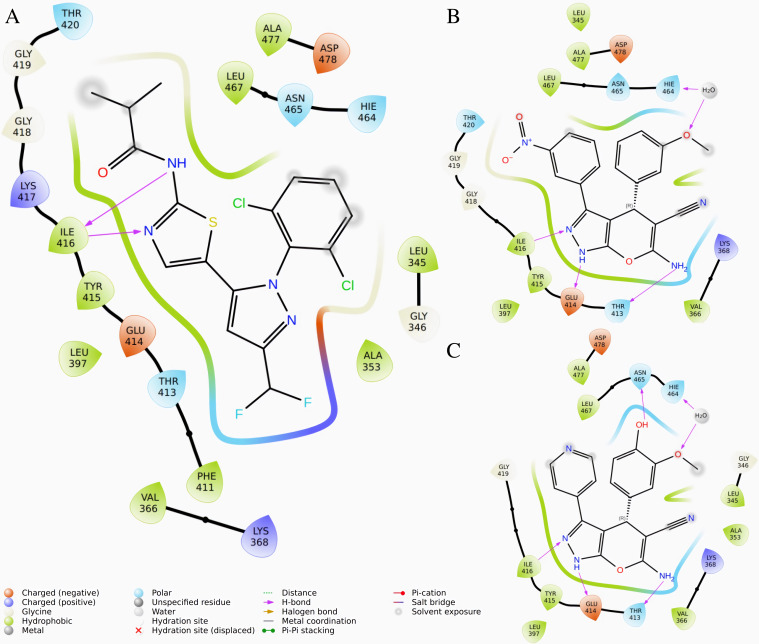
2D Interaction Diagram of Compounds with LIMK1. **(A)** LIMKi3 **(B)** HIT100844099 **(C)** HIT103203505.

In order to characterize the binding interactions between LIMKi3, HIT100844099, HIT103203505 and LIMK1, we systematically examined their molecular interactions with key binding pocket residues, with detailed findings visualized in Fig 4 LIMKi3 primarily forms a hydrogen bond with ILE-416, which appears to be central to its binding. HIT100844099, however, not only retains this interaction but also establishes additional hydrogen bonds with GLU-414 and THR-413, as well as forming a water-mediated interaction with PHE-464. This more extensive interaction network suggests enhanced binding stability. HIT103203505 exhibits a similar pattern, engaging in interactions with GLU-414, THR-413, and ASN-465, while also forming a water bridge with PHE-464.

Despite their additional interactions, neither HIT100844099 nor HIT103203505 shows binding at the active site residue ASP-460, which is consistent with LIMKi3’s interaction pattern. This suggests that interactions at ASP-460 may not be essential for binding in this context.

In conclusion, HIT100844099 and HIT103203505 strengthen their binding to LIMK1 by forming additional hydrogen bonds and water-mediated interactions beyond those of LIMKi3. These enhanced interactions suggest that they may offer improved binding stability and specificity, making them promising candidates for further investigation as LIMK1 inhibitors. The absence of interactions at ASP-460 across all compounds indicates that this residue is likely not critical for their binding efficacy.

### 3.5. Binding stability evaluation of LIMKi3 HIT103203505 and HIT100844099 with LIMK1 through molecular dynamics simulations

**Fig 5:**
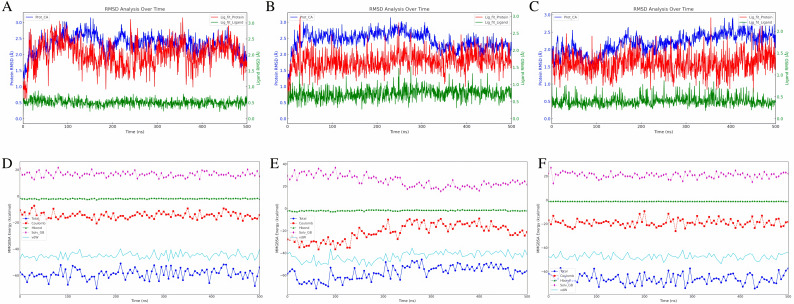
Binding Stability and Free Energy Calculations of the Three Compounds. Dynamic changes in RMSD over time: **(A)** LIMKi3 **(B)** HIT100844099 **(C)** HIT103203505. Dynamic changes in MMGBSA over time: **(D)** LIMKi3 **(E)** HIT100844099 **(F)** HIT103203505.

To evaluate the binding stability of LIMKi3, HIT103203505, and HIT100844099 with LIMK1, we conducted 500 ns molecular dynamics simulations. RMSD analysis (Figs 5A–C) highlighted different stability levels among the complexes. LIMKi3-LIMK1 stabilized between 1.8 and 2.0 Å, indicating early equilibrium. HIT103203505-LIMK1 exhibited greater fluctuations (2.0 to 2.4 Å), while HIT100844099-LIMK1 showed the highest stability with minimal fluctuations (1.5 to 1.7 Å). Ligand stability in the binding pocket followed a similar trend, with LIMKi3 displaying fluctuations between 2.0 and 2.5 Å, HIT103203505 showing tighter binding (1.2 to 1.6 Å), and HIT100844099 demonstrating the most stable binding (1.0 to 1.2 Å). The internal ligand structures were also stable, with HIT100844099 exhibiting the lowest internal RMSD (0.3 to 0.4 Å), while LIMKi3 and HIT103203505 showed slightly higher values.

The energy analysis (Figs 5D–F) showed that HIT103203505 had the strongest binding, with a total binding energy of -65.95 kcal/mol, surpassing LIMKi3 (-58.78 kcal/mol) and HIT100844099 (-57.42 kcal/mol). Coulomb interactions were most favorable for HIT100844099 (-21.07 kcal/mol) and HIT103203505 (-18.97 kcal/mol), both better than LIMKi3 (-14.49 kcal/mol). LIMKi3, however, displayed the strongest hydrogen bond energy (-1.77 kcal/mol), while HIT100844099 and HIT103203505 showed weaker hydrogen bonding (-1.64 kcal/mol and -1.02 kcal/mol, respectively). For solvent polarization, HIT100844099 (24.74 kcal/mol) and HIT103203505 (20.88 kcal/mol) surpassed LIMKi3 (16.66 kcal/mol), indicating stronger solvent interactions. In terms of van der Waals interactions, HIT103203505 had the lowest energy (-47.44 kcal/mol), indicating stronger binding compared to LIMKi3 (-44.83 kcal/mol) and HIT100844099 (-43.15 kcal/mol).

Overall, HIT103203505 exhibited the most favorable binding performance across most parameters, while HIT100844099 displayed strong Coulomb and solvent interactions but slightly weaker total binding and van der Waals interactions compared to LIMKi3.

### 3.6 Comparative analysis of interaction modes and hydrogen bond dynamics of LIMKi3 HIT103203505 and HIT100844099 with LIMK1

**Fig 6:**
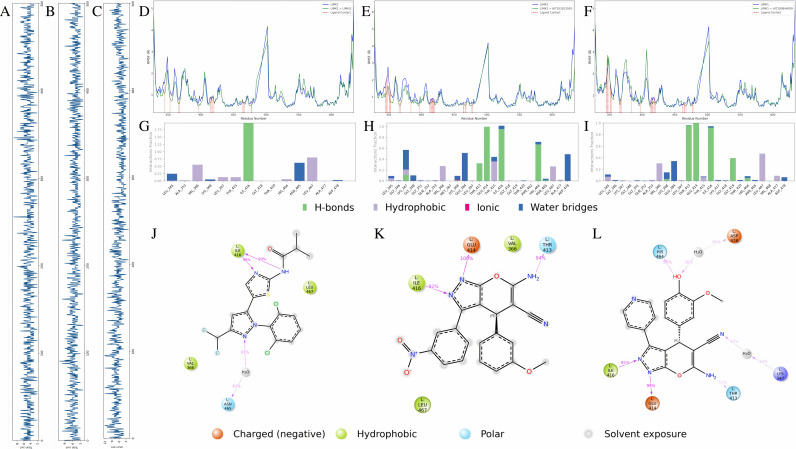
Interaction Analysis of the Three Compounds with LIMK1. Dynamic changes in the number of hydrogen bonds over time: **(A)** LIMKi3 **(B)** HIT100844099 **(C)** HIT103203505. Impact on RMSF of LIMK1 before and after binding: **(D)** LIMKi3 **(E)** HIT100844099 **(F)** HIT103203505. Interaction statistics of compounds with different residues: **(G)** LIMKi3 **(H)** HIT100844099 **(I)** HIT103203505. Binding mode of compounds with LIMK1: **(J)** LIMKi3 **(K)** HIT100844099 **(L)** HIT103203505.

After completing the stability analysis, this section focuses on a comparative analysis of the interaction modes of the three compounds. First, we analyzed the number of hydrogen bonds, as shown in Figs 6A–C. The hydrogen bond count for all three compounds reached a dynamic equilibrium, with LIMKi3 maintaining approximately five hydrogen bonds with slight fluctuations, HIT103203505 stabilizing around six, and HIT100844099 exhibiting the highest stability with around eight hydrogen bonds.

All three compounds (LIMKi3, HIT103203505, and HIT100844099) impacted the ATP binding site (residues 345–353 and 368) and the active site (residue 460) of LIMK1. Overall, these compounds reduced the flexibility of these critical sites, as indicated by a decrease in RMSF values, as shown in Figs 6D–F, suggesting increased structural stability in these regions. Particularly, at the 345–353 residues, the effects of all three compounds were similar, indicating enhanced stability at the ATP binding site upon compound binding. However, there were also differences among the compounds. HIT103203505 and LIMKi3 showed similar effects in significantly stabilizing the ATP binding site and the active site. In contrast, although HIT100844099 reduced the RMSF values across most regions, it caused a slight increase in flexibility at residue 368, as reflected by a higher RMSF value at that site. This suggests that HIT100844099 may modulate LIMK1 differently than the other two compounds, displaying more flexibility at certain ATP binding sites.

Next, we analyzed the interaction patterns of the three compounds with different LIMK1 residues during the simulation, as shown in Figs 6G–I. Compared to LIMKi3, which binds tightly in the co-crystal structure, the interaction sites for HIT103203505 and HIT100844099 were more dispersed, indicating some degree of conformational change during the binding process. As shown in Figs 6J–L, both HIT100844099 and HIT103203505 featured groups that were not fully engaged with LIMK1 residues, allowing for some flexibility in binding. Notably, LIMKi3 also displayed groups that did not interact with any residues. Consequently, we plan to conduct scaffold hopping based on the structure of HIT100844099 in future studies, aiming to design a potential inhibitor with enhanced stability and stronger binding to LIMK1.

### 3.7. Scaffold hopping stability analysis and site-directed mutagenesis validation of S11a0000168202 compared to HIT100844099 in binding to LIMK1

**Fig 7:**
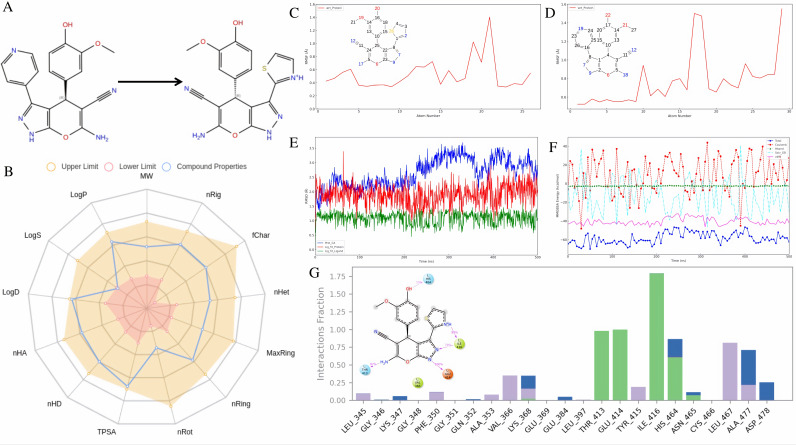
Post-Modifications Evaluation and Interaction Analysis of S11a-0000168202. **(A)** Differences before and after scaffold hopping of HIT100844099. **(B)** Medicinal Chemistry Radar Chart of S11a-0000168202. RMSF differences before and after scaffold hopping for binding with LIMK1: **(C)** S11a-0000168202 **(D)** HIT100844099. **(E)** Dynamic changes in RMSD of S11a-0000168202 over time. **(F)** Dynamic changes in MMGBSA of S11a-0000168202 over time. **(G)** Interaction type statistics of S11a-0000168202 with LIMK1.

Based on the previously obtained binding mode of HIT100844099 with LIMK1, we performed scaffold hopping on the groups that did not interact with any amino acids, followed by visual screening. This process led to the selection of S11a-0000168202. The differences between the two compounds are shown in Fig 7A: in S11a-0000168202, a carbon atom in the ring of HIT100844099 was replaced by a sulfur atom, forming a thiazole ring, and stereochemical features were introduced at that position.

The physicochemical properties of S11a-0000168202 were analyzed (Fig 7B and S1 Table). The compound has a molecular weight of 368.08, a volume of 343.18, and a density of 1.073. It contains eight hydrogen bond acceptors [32] and five hydrogen bond donors (nHD), with three rotatable bonds and four rings, the largest of which consists of nine atoms. The compound has nine heteroatoms (nHet), a charge of + 1, 22 rigid atoms, and a flexibility index of 0.136, with one stereocenter. The total polar surface area (TPSA) is 131.32, while its water solubility (logS) is -4.126, and its partition coefficient (logP) is 2.401, with a distribution coefficient (logD) of 2.869, indicating moderate lipophilicity and solubility.

To highlight the advantages of S11a-0000168202, molecular dynamics simulations were conducted under similar conditions. As shown in the RMSF data (Figs 7C–D), S11a-0000168202 displayed lower fluctuations compared to its precursor HIT100844099, indicating greater stability when bound to the target protein. The RMSF curve of S11a-0000168202 shows reduced flexibility, particularly at key residues, reflecting tighter binding. In contrast, HIT100844099 exhibited higher RMSF values, indicating greater flexibility and lower stability. The RMSF data thus support the improved binding stability of S11a-0000168202 following sulfur substitution and stereochemical modification.

The RMSD and MMGBSA data further illustrate the binding stability and binding energy of S11a-0000168202 with the target protein, as shown in Figs 7E–F. The RMSD of the protein Cα atoms (Prot_CA) increased gradually and stabilized between 1.5 and 1.8 Å, indicating good protein backbone stability throughout the simulation. The ligand’s RMSD relative to the protein (Lig_wrt_Protein) reached a maximum of 2.6 Å but remained mostly between 1.1 and 1.8 Å, indicating some adjustment within the binding site but overall stable binding. The internal RMSD of the ligand (Lig_wrt_Ligand) remained between 0.5 and 1.0 Å, reflecting minimal conformational changes and structural stability.

The MMGBSA analysis revealed that the total binding free energy (r_psp_MMGBSA_dG_Bind) of S11a-0000168202 with the target protein stabilized between -60 and -67 kcal/mol, indicating strong binding. Van der Waals interactions (r_psp_MMGBSA_dG_Bind_vdW), the main contributing factor, remained between -40 and -44 kcal/mol, highlighting the importance of hydrophobic interactions. Coulomb interactions (r_psp_MMGBSA_dG_Bind_Coulomb) and solvation energy (r_psp_MMGBSA_dG_Bind_Solv_GB) showed some fluctuation during the simulation but contributed to overall binding stability.

In summary, RMSD data demonstrated the structural stability of S11a-0000168202 when bound to the protein, with the ligand maintaining a stable conformation and binding pose. MMGBSA data supported the strong binding of S11a-0000168202 through van der Waals and electrostatic interactions. Together, these results indicate that S11a-0000168202 exhibits excellent binding stability and strong affinity for the target protein.

In the subsequent interaction analysis (Fig 7G), S11a-0000168202 predominantly forms hydrogen bonds with the backbone atoms of GLU-414, ILE-416, and HIS-464, as well as with the side chain of THR-413. Additionally, it engages in water-mediated interactions with VAL-366 and LEU-467. To confirm the significance of these interactions in the binding of S11a-0000168202 to LIMK1, we performed site-directed mutagenesis by replacing the four key amino acids involved in hydrogen bond formation with alanine. Alanine scanning was chosen because alanine is a small, non-reactive residue that effectively removes side chain interactions while minimizing changes to the protein’s overall structure. This approach allows for the assessment of each residue’s specific contribution to binding.

**Fig 8:**
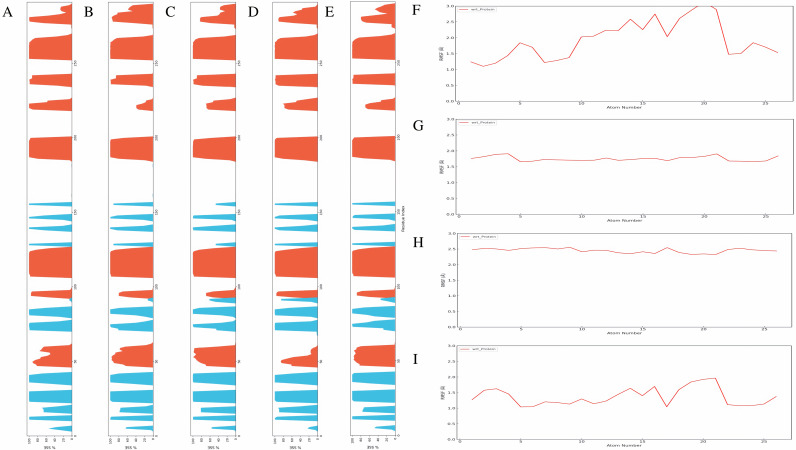
Site-Directed Mutagenesis Validation of the Structure-Activity Relationship of S11a-0000168202. Secondary structure analysis of LIMK1: (A) WT **(B)** T413A **(C)** E414A **(D**) I416A **(E)** H464A. RMSF of S11a-0000168202 bound to LIMK1 after mutations: **(F)** T413A **(G)** E414A **(H)** I416A **(I)** H464A.

To ensure that these mutations did not induce significant structural changes, we first analyzed the secondary structure of LIMK1 during the simulation. As shown in Figs 8A–E, the introduction of these alanine mutations did not cause notable alterations in the secondary structure compared to the wild type, indicating that subsequent changes in binding stability are directly related to the mutated residues rather than overall structural changes.

We then analyzed the RMSF of S11a-0000168202 bound to the four mutated forms of LIMK1 (Figs 8F–I). The RMSF results revealed significant differences compared to the wild type, suggesting that these four residues are critical for the binding of S11a-0000168202 to LIMK1. The observed increase in RMSF values in the mutated forms indicates a reduction in binding stability, highlighting the importance of these specific residues in maintaining effective ligand interaction.

## 4. Discussion

This study focused on the design, optimization, and evaluation of LIMK1 inhibitors, aiming to develop more effective treatments for gastric cancer. LIMK1 plays a crucial role in cytoskeletal dynamics, influencing cell migration, invasion, and tumor progression, especially in aggressive forms of cancer such as gastric cancer [33]. Among the known LIMK1 inhibitors, LIMKi3, an ATP-competitive LIMK1/2 inhibitor, has demonstrated promising anti-tumor activity in vitro [22]. However, its clinical translation faces several significant challenges, including suboptimal selectivity between LIMK1 and LIMK2, potential off-target effects on related kinases such as AMPKα2, and poor aqueous solubility that affects bioavailability. Additionally, concerns regarding toxicity due to the widespread expression of LIMKs in various tissues remain a major hurdle. Furthermore, long-term treatment with LIMKi3 may lead to resistance through compensatory signaling pathways. These limitations underscore the urgent need for the development of next-generation LIMK inhibitors with improved selectivity, solubility, and safety profiles, providing more effective therapeutic options for gastric cancer patients.

The exploration of LIMKi3, HIT103203505, and HIT100844099 provided insights into the binding modes and interactions with LIMK1, leading to further modifications and improvements in compound design, particularly with S11a-0000168202.

Our initial analysis of LIMKi3, HIT103203505, and HIT100844099 demonstrated that HIT100844099 exhibited promising binding stability due to additional hydrogen bonding and water-mediated interactions [8]. In addition, the bioactivity data for HIT100844099 showed promising inhibitory effects across multiple biological pathways, including its role as a cystinyl aminopeptidase inhibitor [34] (Pa = 0.679, Pi = 0.003) and apoptosis agonist (Pa = 0.562, Pi = 0.031), highlighting its potential therapeutic effects. However, there was room for improvement in binding efficiency and stability, which led to the structural modification of HIT100844099, producing S11a-0000168202. The replacement of a carbon atom with a sulfur atom in the ring structure, forming a thiazole ring and introducing stereochemical features, resulted in improved molecular properties and interaction patterns.

In molecular dynamics simulations, S11a-0000168202 demonstrated greater stability than its precursor HIT100844099, with lower RMSF fluctuations, especially at key residues within LIMK1. This indicates that the compound binds more tightly to the protein, reducing flexibility and enhancing binding stability [35]. Compared to LIMKi3, S11a-0000168202 not only retains the critical hydrogen bond with ILE-416 but also forms additional hydrogen bonds with GLU-414, THR-413, and HIS-464, thereby significantly strengthening the binding network. Furthermore, S11a-0000168202 establishes water-mediated interactions with VAL-366 and LEU-467, contributing to enhanced binding stability. Residues such as GLU-414 and THR-413 play vital roles in LIMK1 activity due to their involvement in maintaining the kinase’s structural and functional integrity. Specifically, GLU-414, located within the ATP-binding site, is crucial for stabilizing ATP during phosphorylation reactions, while THR-413, situated in the activation loop, ensures the proper conformation needed for substrate recognition and catalytic activity. Phosphorylation of residues like THR-508 in LIMK1’s activation loop is also essential for its activation by upstream kinases, such as ROCK and PAK. These phosphorylation events directly influence LIMK1’s ability to phosphorylate its primary substrate, cofilin, thereby regulating actin dynamics and key cellular processes like migration and invasion. These structural insights underscore the indispensable roles of these residues in LIMK1’s function and validate their biological relevance as potential targets for designing selective LIMK inhibitors. RMSD and MMGBSA analyses further confirmed that S11a-0000168202 maintained stable binding throughout the simulation, driven by strong van der Waals interactions and favorable electrostatic contributions. Collectively, these findings highlight its potential as a highly robust LIMK1 inhibitor [36].

Although the newly identified compounds demonstrated low structural similarity to LIMKi3, this divergence may offer both opportunities and challenges. On one hand, novel scaffolds can enable new binding interactions, potentially improving selectivity toward LIMK1. On the other hand, these structural differences could also introduce unintended interactions with off-target proteins, especially other kinases sharing conserved ATP-binding pockets. While our current analysis focused on LIMK1, future studies will integrate off-target prediction tools (e.g., Swiss Target Prediction) and comparative docking against related kinases such as LIMK2, ROCK1, and PAK1. These approaches will be essential to evaluate compound specificity and reduce the risk of adverse off-target effects during therapeutic development.

To better characterize the binding dynamics and overcome potential limitations of conventional molecular dynamics (MD) simulations, several enhanced sampling methods have been developed, including WESTPA [44], DeepWEST [45], Ligand-GaMD [46], GaMD-WE [47], metadynamics [48], and SEEKR [49]. These approaches are capable of accelerating the sampling of rare events and conformational transitions by overcoming high energy barriers. However, in the present study, these techniques were not implemented due to the requirement of advanced computational infrastructure and specialized methodological setups, which were not available at the time of analysis. While simplified alternatives such as accelerated MD (aMD) or umbrella sampling may be more accessible, their integration requires pre-defined collective variables and parameter optimization that exceed the current scope. Despite these constraints, the 500 ns conventional MD simulations conducted in this work exhibited early RMSD convergence, stable hydrogen bond formation, and consistent MMGBSA profiles. These indicators suggest that key conformational states relevant to ligand binding were sufficiently captured for the intended mechanistic evaluations. Future investigations will consider incorporating enhanced sampling protocols to further extend the conformational space exploration.

In addition to these stability improvements, S11a-0000168202 displayed favorable ADMET properties. In terms of absorption, although it shows suboptimal Caco-2 permeability, indicating moderate challenges in crossing the intestinal epithelial barrier [37], its human intestinal absorption (HIA)cc prediction is favorable, suggesting effective uptake in the gastrointestinal tract [38]. It also exhibits low inhibition of P-glycoprotein, reducing concerns over efflux-related issues, although being a P-glycoprotein substrate may affect its bioavailability [39].

For distribution, the compound shows high plasma protein binding (PPB), with over 95% of the drug expected to bind to plasma proteins, which could limit the amount of free drug available to reach its target [40]. Despite this, the fraction of unbound drug remains within an acceptable range, allowing for a pharmacologically active concentration. The compound’s moderate potential to cross the blood-brain barrier suggests it could access the central nervous system if required for therapeutic purposes [41].

Regarding metabolism, S11a-0000168202 shows interaction with several cytochrome P450 enzymes, particularly as an inhibitor of CYP1A2, CYP2C19, and CYP3A4. This raises the potential for drug-drug interactions, as the compound could interfere with the metabolism of other medications [42]. Additionally, it is a substrate for several enzymes, indicating that it will likely undergo metabolic processing, which may influence its clearance and bioavailability.

In terms of excretion, the compound is predicted to have moderate clearance and a relatively short half-life. This could necessitate frequent dosing or formulation modifications to ensure sustained therapeutic levels.

From a toxicity perspective, S11a-0000168202 displays a favorable profile with a low risk of cardiotoxicity, as indicated by its minimal probability of blocking the hERG channel [43–49]. However, concerns arise regarding its potential hepatotoxicity and high risk of drug-induced liver injury (DILI), as well as its high probability of causing acute oral toxicity and respiratory toxicity. While it does not exhibit significant carcinogenic risks, some caution is needed regarding its potential for skin sensitization.

Despite the above ADMET trade-offs, S11a-0000168202 was prioritized for further development due to its superior binding stability, robust interactions with key LIMK1 residues (GLU-414, THR-413, ILE-416), and improved molecular dynamics behavior relative to its precursor HIT100844099 and other screened candidates. Comparative analysis showed that while certain toxicity risks were predicted, the compound retained favorable characteristics in terms of aqueous solubility, polar surface area, and compliance with established drug-likeness rules (e.g., Pfizer, GSK, Rule of Three). These properties supported its selection for subsequent structure-activity relationship (SAR) optimization.

Although no formal multiparametric scoring model was applied in this study, the selection of S11a-0000168202 was based on a qualitative integration of MMGBSA binding affinity, ligand RMSD/RMSF convergence, interaction persistence, and pharmacokinetic parameters. Future prioritization workflows may benefit from adopting desirability scoring or machine learning-based ADMET-balance models to quantify trade-offs between potency and safety.

In addition, although MOF/COF-based drug delivery systems were conceptually proposed in the conclusion section, the present study did not explore structure-level strategies to attenuate toxicity. Moving forward, in silico redesign of problematic substructures—such as minimizing H-bond donors near metabolic hotspots, introducing polar substitutions, or modifying planarity—will be considered to reduce CYP450 inhibition and off-target liabilities. These modifications will form the basis for future lead refinement cycles.

To facilitate translational progress, we also outline our planned experimental validation: (i) in vitro assays will be conducted in gastric cancer cell lines AGS and MKN-45 using CCK-8, colony formation, and migration assays, along with Western blot to assess LIMK1 and cofilin signaling; (ii) in vivo studies may utilize an MKN-45 xenograft mouse model and PK testing in BALB/c mice; (iii) collaborations with pharmacology labs are underway to support synthesis, testing, and animal studies.

However, despite the encouraging results from computational studies, it is important to note that the findings presented here lack experimental validation. While molecular dynamics simulations and binding energy calculations provide valuable insights into the potential interactions and stability of these compounds, these predictions must be tested in biological and biochemical assays to confirm their relevance in a physiological context. In vitro and in vivo experiments will be essential to verify the binding affinities, inhibitory effects, and pharmacokinetic profiles of S11a-0000168202 and other candidate compounds. Such experiments would provide critical data on the selectivity, efficacy, and safety of these inhibitors, which cannot be fully predicted by computational models alone.

In conclusion, the design and optimization of S11a-0000168202 represent significant advancements in the development of LIMK1 inhibitors. The improved binding stability, ADMET profile, and molecular properties of this compound, combined with its interaction profile, highlight its potential as a therapeutic agent for gastric cancer. However, the lack of experimental validation presents a key limitation in this study. Future work should focus on experimentally confirming the computational predictions through in vitro assays and animal studies to assess the true therapeutic potential of these compounds.

## Conclusion

This study developed and optimized S11a-0000168202 as a promising LIMK1 inhibitor, showing significant improvements in binding stability and molecular interactions compared to HIT100844099. The compound demonstrated strong binding to key LIMK1 residues such as GLU-414, ILE-416, and HIS-464, while also exhibiting favorable ADMET properties, including moderate lipophilicity, good human intestinal absorption, and low P-glycoprotein inhibition.Despite predicted risks such as CYP2D6 inhibition and hepatotoxicity, S11a-0000168202 was prioritized due to its superior stability, interaction pattern, and drug-likeness compliance. These trade-offs were qualitatively balanced, and future efforts will address toxicity via scaffold redesign and molecular refinement.Additionally, we outline a clear experimental roadmap for validation. In vitro studies in AGS and MKN-45 cell lines and potential xenograft models will confirm efficacy and toxicity. Collaborations with experimental groups have been initiated to accelerate compound translation.Overall, this compound holds strong potential as a LIMK1-targeted therapeutic agent for gastric cancer, pending further experimental validation.

## Supporting information

S1 Fig Medicinal Chemistry Radar Chart. (A) HIT102568167 (B) HIT103799421 (C) HIT101601533 (D) HIT100835212 (E) HIT105357965 (F) HIT102689075 (G) HIT107134831 (H) HIT102031352 (I) HIT101337631.
(JPEG)

S1 Table ADMET profile of HIT100844099. (CSV)

S2 Table ADMET profile of S11a-0000168202. (CSV)
