# Supplementary figures and images for: Design, optimization, and ADMET evaluation of S11a-0000168202: A promising LIMK1 inhibitor for gastric cancer treatment
Source: PLoS One. 2025 May 14;20(5):e0323699. doi: 10.1371/journal.pone.0323699 (PMC12077675; doi:10.1371/journal.pone.0323699)

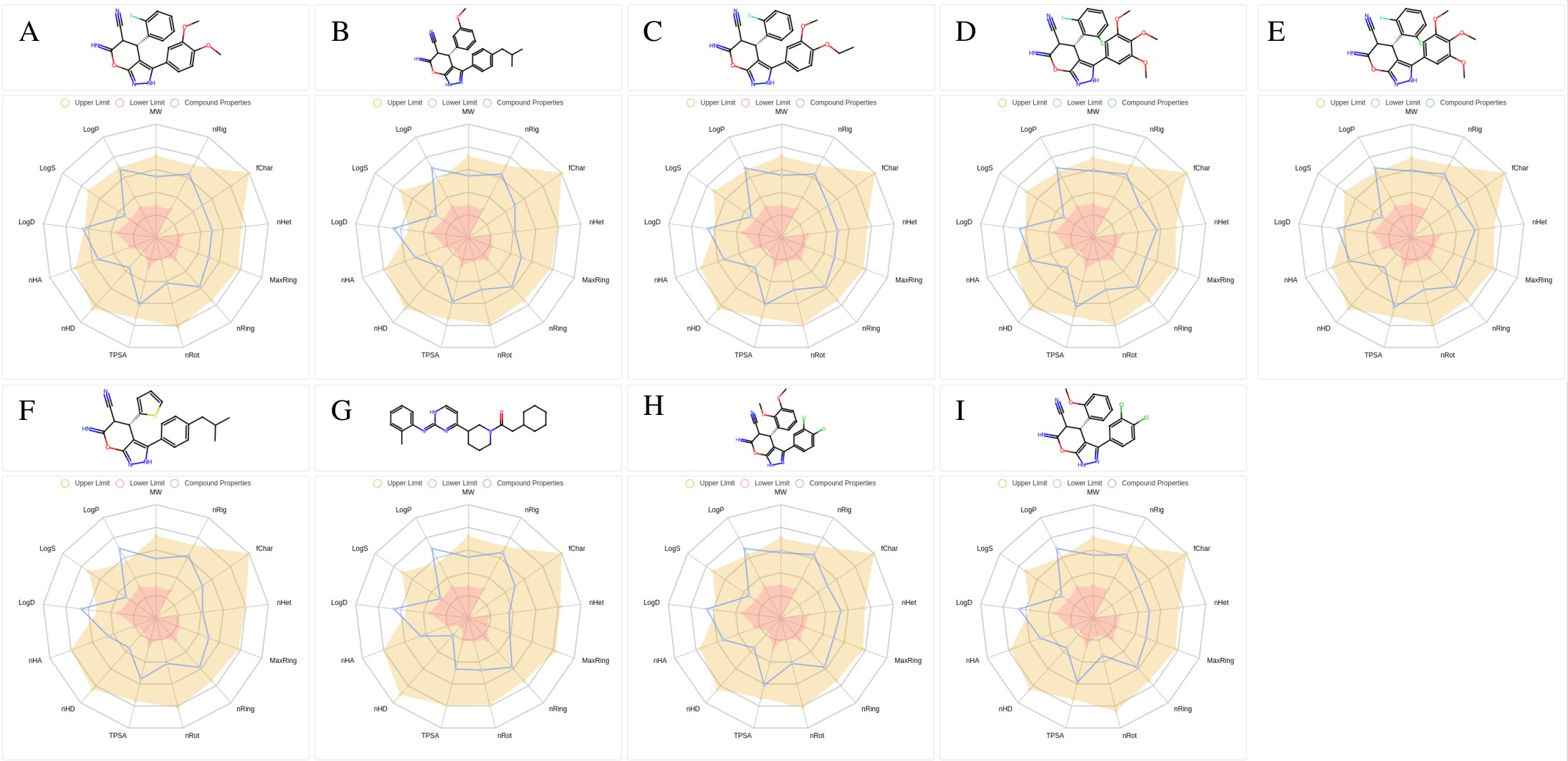

Supplement: S1 Fig — (A) HIT102568167 (B) HIT103799421 (C) HIT101601533 (D) HIT100835212 (E) HIT105357965 (F) HIT102689075 (G) HIT107134831 (H) HIT102031352 (I) HIT101337631. (JPEG) [file pone.0323699.s001.JPEG]
